# Cloning the Horse RNA Polymerase I Promoter and Its Application to Studying Influenza Virus Polymerase Activity

**DOI:** 10.3390/v8060119

**Published:** 2016-05-31

**Authors:** Gang Lu, Dong He, Zengchao Wang, Shudan Ou, Rong Yuan, Shoujun Li

**Affiliations:** 1 College of Veterinary Medicine, South China Agricultural University, Guangzhou 510000, China; 18814113742@163.com (G.L.); donghe9108@163.com (D.H.); lugang19860501@163.com (Z.W.); whj751364486@163.com (S.O.); jeremyjessica@163.com (R.Y.); 2 Key Laboratory of Comprehensive Prevention and Control for Severe Clinical Animal Diseases of Guangdong Province, Guangzhou 510000, China

**Keywords:** RNA polymerase I promoter, equine influenza virus, polymerase activity

## Abstract

An influenza virus polymerase reconstitution assay based on the human, dog, or chicken RNA polymerase I (PolI) promoter has been developed and widely used to study the polymerase activity of the influenza virus in corresponding cell types. Although it is an important member of the influenza virus family and has been known for sixty years, no studies have been performed to clone the horse PolI promoter or to study the polymerase activity of equine influenza virus (EIV) in horse cells. In our study, the horse RNA PolI promoter was cloned from fetal equine lung cells. Using the luciferase assay, it was found that a 500 bp horse RNA PolI promoter sequence was required for efficient transcription. Then, using the developed polymerase reconstitution assay based on the horse RNA PolI promoter, the polymerase activity of two EIV strains was compared, and equine myxovirus resistance A protein was identified as having the inhibiting EIV polymerase activity function in horse cells. Our study enriches our knowledge of the RNA PolI promoter of eukaryotic species and provides a useful tool for the study of influenza virus polymerase activity in horse cells.

## 1. Introduction

The genome of the influenza A virus consists of eight negative-sense single-stranded viral RNAs (vRNAs). The viral replication and transcription cycle is initiated by viral ribonucleoproteins (vRNPs), which include the nucleoprotein (NP) and the three subunits of the polymerase complex (PB1, PB2, and PA) [1]. Neumann *et al.* and Fodor *et al.* established a reverse genetics system for the influenza virus based on the human RNA polymerase I (PolI) promoter [2,3]. In the system, each viral cDNA is inserted between the human RNA PolI promoter and the RNA PolI terminator. These plasmids, together with four other eukaryotic expression plasmids encoding NP and polymerases, are transfected into 293T or Vero cells. The synthesized RNPs transcribe the (−)vRNAs into mRNAs and (+)cRNAs, and the (+)cRNAs serve as templates to generate more vRNAs [1,2,3,4]. In this process, the human RNA PolI promoter is recognized by the human RNA PolI. The RNA PolI promoter is known to be species-specific [5,6,7,8]. The RNA PolI promoter from one species may not be recognized by RNA PolI from distantly-related species. To date, in addition to the human-derived RNA PolI promoter, RNA PolI promoters from the chicken, dog, and mouse have also been cloned and applied to efficiently rescue the influenza virus [5,6,7,9].

The influenza virus polymerase reconstitution assay has been widely applied to investigate influenza virus polymerase activity *in vitro* [10,11,12,13]. In this assay, an artificial influenza virus-like reporter gene, flanked by 5′ and 3′ terminal non-coding sequences of one segment of the influenza virus, is inserted into a reporter plasmid under the control of the PolI promoter. This reporter plasmid, together with plasmids expressing PB1, PB2, PA, and NP proteins of the influenza virus, is co-transected into susceptible cells. The PolI promoter directs the synthesis of negative-sense, virus-like vRNA from the reporter gene and the positive-sense mRNA is further synthesized by viral polymerase using vRNA as a template. By detecting the reporter protein level, the influenza virus polymerase activity can be identified indirectly in the influenza virus polymerase reconstitution assay. Until now, the influenza virus polymerase reconstitution assay has been the most widely used method to estimate the influenza virus polymerase activity *in vitro*. Using this method, it has been identified that some amino acid sites in NP and polymerases are important for viral replication or host adaption during evolution [10,14,15]. However, due to the species-specific characteristic of the RNA PolI promoter, it is necessary to clone the RNA PolI promoter from the corresponding host cells before investigating the influenza virus polymerase activity in the host. The cloned human, chicken and dog RNA PolI promoter has been applied for studying influenza virus polymerase activity in the corresponding cell types [5,7,10,11,12,16].

Equine influenza virus (EIV) can cause acute and highly-contagious disease in the horse species and is responsible for equine influenza outbreaks in horses [17,18]. Two subtypes of EIV (H7N7, H3N8) have been identified in the horse population since 1956 [19]. The H7N7 subtype EIV has not been isolated since 1979, and the H3N8 subtype EIV has evolved into three main clades: the pre-divergence clade, the American clade, and the European clade [19,20]. Although EIV has been known for sixty years and is distributed worldwide, the polymerase activity of EIV has not been described in cells. The polymerase activity of the influenza viruses of other species has also not been investigated in horse cells. To date, no research studies on the horse RNA PolI promoter have been reported. Accordingly, the horse RNA PolI promoter was cloned in our study and was used to estimate influenza virus polymerase activity in horse-derived cells.

## 2. Results and Discussion

**Figure 1:**
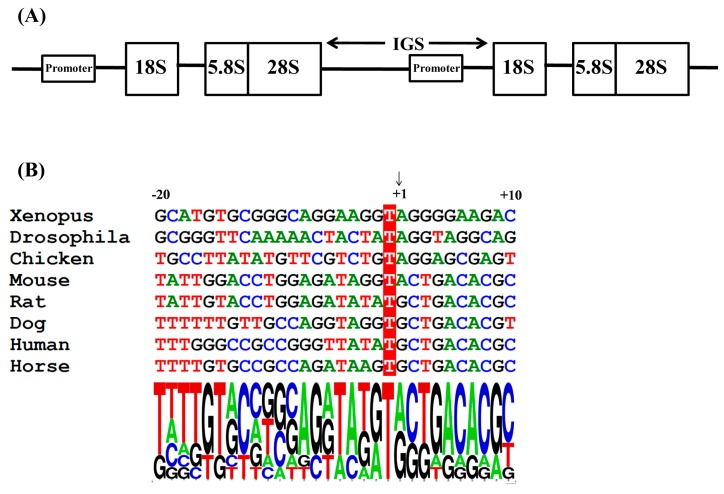
Molecular mapping of the horse RNA PolI promoter. (**A**) Map of horse ribosomal DNA: 5.8S, 18S, and 28S rRNAs are in clusters of head-to-tail repeats. The intergenic spacer (IGS) region is located between the 28S and 18S rRNA coding sequences and contains the horse RNA PolI promoter sequence; and (**B**) alignment of the sequences around the RNA PolI transcription initiation site of horse and other species. The transcription initiation site is indicated by an arrow and labeled as +1. The conserved −1 position (T) is shown with a red background. The base frequency around the RNA PolI transcription initiation site is identified using the WebLOGO tool [25].

In higher eukaryotes, the 45S ribosomal RNA (rRNA) comprises the 5.8S, 18S, and 28S rRNAs in clusters of head-to-tail repeats (Figure 1A). The intergenic spacers (IGS) between the 28S and 18S rRNA coding sequences contain the RNA PolI promoter sequence and other regulatory elements [8,21]. The transcription initiation site of the RNA PolI promoter is located upstream of the 18S rRNA, with a size of 3000–5000 bp [7]. To clone the horse RNA PolI promoter sequence, the horse 18S rRNA nucleotide sequence (GenBank accession no. NR_046271.1) was identified in the *Equus caballus* genome database [22]. Then, the obtained horse 18s rRNA nucleotide sequence was queried using the Blastn search tool in the ensembl database [23], and the chromosome sequence Un0592: 1-41,063 that contained the predicted horse 18s rRNA gene was found. Previous research has determined that the nucleotide residues around the transcription initiation site are highly conserved among eukaryotes (Figure 1B) [6,7]. Accordingly, a homology search was performed on Un0592: 1–41,063 to find the possible RNA PolI transcription initiation site using Bioedit software (Version 7.0.9.0) [24]. Finally, the nucleotide positions from 8755 to 8784 were identified as having the most similarity with the RNA PolI transcription initiation sites of other eukaryotes. The first base of the predominant RNA transcript of the indicated species was referred to as +1 in our study (Figure 1B). Among all eukaryote species, the −1 position was always T, and the +1 position was A or G. Among the analyzed mammal species, the nucleotide sequence from the +2 to +8 positions (CTGACACG) was constant; the −2 position was A or G, and the −4, −5, and −7 positions were A, T, and G, respectively.

**Figure 2:**
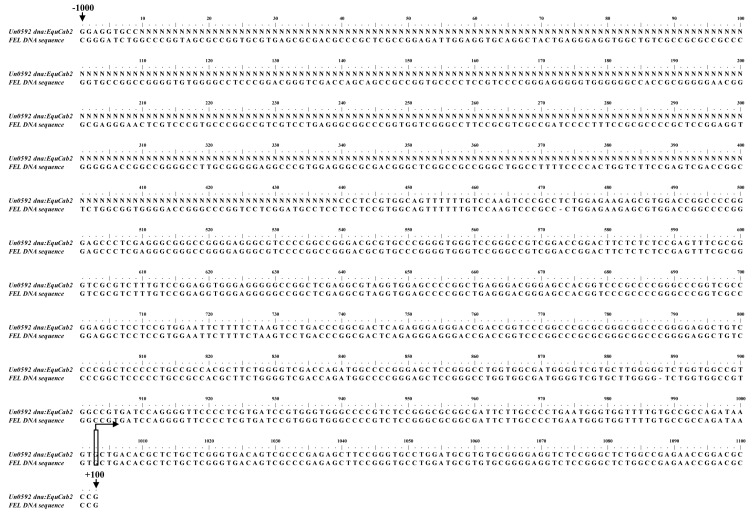
Alignment of the horse genomic DNA sequence (Un0591) and the cloned genomic DNA sequence from FEL cells in our study. The transcription initiation site is labeled as +1. The −1000 and +100 positions of the horse RNA PolI transcription initiation site are shown by arrows.

Research on human-, canine-, chicken-, and mouse-derived RNA PolI indicated the narrowest sequences required for the highest RNA PolI promoter activity was no more than 500 bp [2,3,4,5,7]. One primer pair targeting from −2146 to +460 of the predicted horse RNA PolI transcription initiation site was designed using Oligo 7.0 software (2146-F, GTCTGTTGCCCATGTTCCTCAG; 460-R, ACATCGATCAGCAGCATAACGC). The genomic DNA was extracted from fetal equine lung (FEL) cells, and the target DNA fragment was amplified by standard PCR and then sequenced (BGI, Guangzhou, China). The obtained genomic sequence of the FEL cells was aligned with the horse genomic DNA sequence (from nt −1000 to +100 of the predicted horse RNA PolI transcription initiation site). The result indicated that except for the undetermined bases in the horse genomic DNA sequence available online (shown as N in Figure 2), they have a similarity of 99.7%, with some base deletions and insertions in the cloned FEL cell DNA sequence (Figure 2).

**Figure 3:**
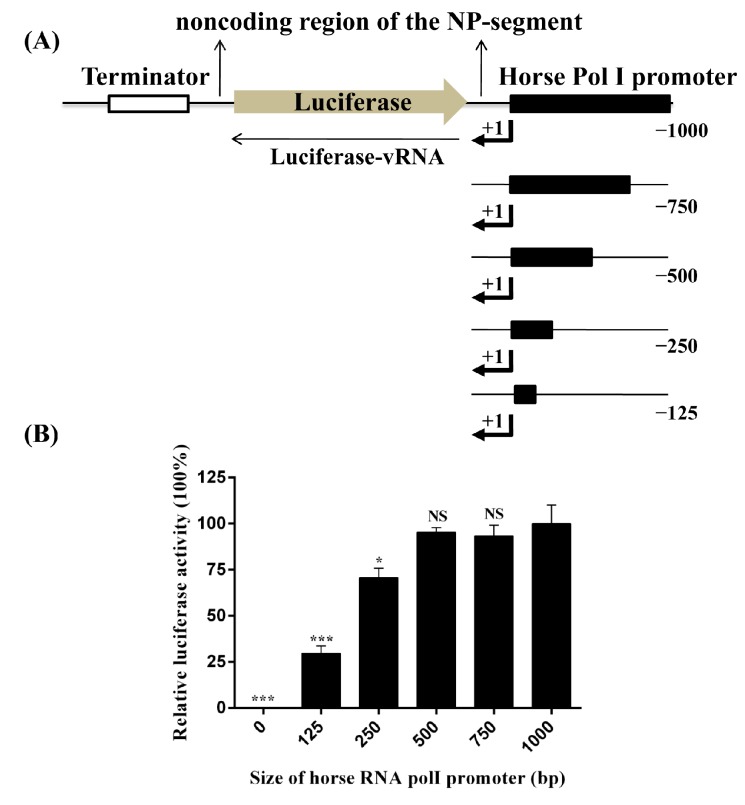
Determination of the transcription efficiency of horse RNA PolI promoters of different lengths. (**A**) Generation of virus-like luciferase reporter RNAs under the control of horse PolI promoters of various lengths. The antisense firefly luciferase coding sequence was flanked by the 5′ and 3’ noncoding sequences of the NP segment of A/equine/Maimi/1/1963; and (**B**) each of the constructs (100 ng) pLW1000-NPluci, pLW750-NPluci, pLW500-NPluci, pLW250-NPluci, pLW125-NPluci, and pLW0-NPluci was individually combined with pCAGGs-Miami PB1, PB2, PA, NP (100 ng of NP, 50 ng [each] of PB1, PB2, and PA), and pRL-TK (10 ng) and co-transfected into FEL cells in a 24-well plate. At 24 h after transfection, the relative luciferase activity was identified by the dual-luciferase assay. The *Renilla* luciferase value was used to normalize the transfection efficiencies. The relative luciferase activity associated with the pLW1000-NPluci construct was set as 100%. Each experiment was repeated three times. Statistical significance was compared with the result of the pLW1000-Npluci group, using an unpaired two-tailed Student’s *t*-test with GraphPad Prism 5.01 [27] (**, *p* < 0.01; ***, *p* < 0.001; NS, not significant).

To determine the transcription efficiency of the horse RNA PolI promoter cloned in our study, the antisense firefly luciferase coding sequence, flanked by the 5′ and 3’ noncoding sequences of the NP segment of A/equine/Miami/1/1963 (Miami/1963, the first H3N8 subtype EIV isolate of the pre-divergence clade) [26], was inserted into the pHW2000 plasmid with the CMV and human PolI promoter removed (the pHW2000 plasmid was kindly provided by Professor Robert G. Webster, St. Jude Children’s Research Hospital). The human PolI promoter was replaced by horse PolI promoters of different lengths (nt −1 to −1000, −750, −500, −250, and −125), and the constructs were designated as pLW1000-NPluci, pLW750-NPluci, pLW500-NPluci, pLW250-NPluci, and pLW125-NPluci, respectively (Figure 3A). One additional construct, pLW0-NPluci, with no horse PolI promoter sequence was also included as a control. The detailed preparation methods of the six constructs are listed in the Supplementary Materials. The six constructs were individually combined with eukaryotic expression plasmids expressing PB1, PB2, PA, and NP proteins of A/equine/Miami/1/1963 (pCAGGs-Miami PB1, PB2, PA, NP, 100 ng of NP, 50 ng [each] of PB1, PB2, and PA) and pRL-TK expressing *Renilla* luciferase (10 ng) and were co-transfected into FEL cells seeded in 24-well plates using ViaFect transfection reagent (Promega, Madison, WI, USA). At 24 h after transfection, the cell lysates were subjected to the dual-luciferase assay (Promega, Madison, WI, USA). The *Renilla* luciferase value was used to normalize the transfection efficiencies. PolI promoter activity was represented as relative luciferase activity, which was calculated as the ratio of firefly luciferase to *Renilla* luciferase. The highest PolI promoter activity was observed for the constructs pLW1000-NPluci, pLW750-NPluci, and pLW500-NPluci (Figure 3B). When the sequence length of the horse PolI promoter was reduced to 250 bp and 125 bp, PolI promoter activity decreased by approximately 30% (*p* < 0.001) and 70% (*p* < 0.01), respectively.

**Figure 4:**
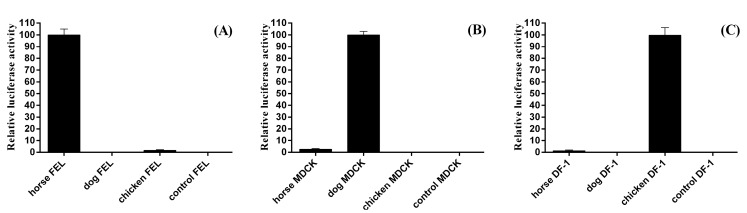
Comparison of transcription efficiency of the horse RNA PolI promoter in FEL, MDCK, and DF-1 cells. The transfection method and the amount of each plasmid was the same as in Figure 2B. The relative luciferase activities associated with the pLW500-NPluci construct in FEL cells (**A**), the pdoPolI-NPluci construct in MDCK cells (**B**) and the pchPolI-NPluci construct in DF-1 cells (**C**) were set as 100%. The relative luciferase activities associated with the pLW0-NPluci construct containing no RNA PolI promoter was used in FEL cells (**A**); MDCK cells (**B**); and DF-1 cells (**C**) as control, respectively. Each experiment was repeated three times.

Next, to compare the transcription efficiency of PolI promoters from different eukaryote species, the 500 bp horse PolI promoter sequence of pLW500-NPluci was replaced with the 457 bp dog (pdoPolI-NPluci) and 425 bp chicken (pchPolI-NPluci) PolI promoter sequences [5,6]. The plasmids pLW500-NPluci, phuPolI-NPluci, pchPolI-NPluci, or pLW0-NPluci (used as control) were individually combined with pCAGGs-Miami PB1, PB2, PA, NP, and pRL-TK and were co-transfected into FEL, 293T, and DF-1 cells in 24-well plates, respectively. The results revealed that the horse RNA PolI promoter has significantly higher transcription efficiency than the human or chicken RNA PolI promoter in FEL cells (Figure 4). Our study further identified the species–specific characteristics of the RNA PolI promoter.

**Figure 5:**
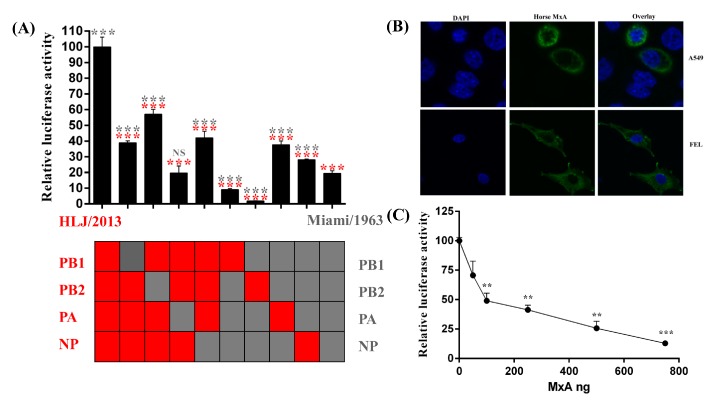
Investigation of EIV polymerase activity (**A**) or the antiviral properties of eqMxA against EIV (**B**,**C**) in FEL cells using our developed influenza virus polymerase reconstitution assay based on the horse PolI promoter. (**A**) Comparison of the polymerase activity of Miami/1963 and HLJ/2013. Each combination of plasmids expressing RNPs of Miami/1963 and HLJ/2013, together with pRL-TK and pLW500-NPluci, was co-transfected separately into FEL cells in 24-well plates. The transfection method and the amount of plasmids was the same as in Figure 2B. The relative luciferase activity associated with HLJ/2013 was set at 100%. Each experiment was repeated three times. The statistical significance of the comparisons between Miami/1963 or HLJ/2013 and other combinations was marked by gray or red, respectively (***, *p* < 0.001; NS, not significant); (**B**) the subcellular location of eqMxA protein with an N-terminal Flag tag in A549 and FEL cells. The pCAGGs-eqMxA plasmid was transfected into A549 or FEL cells. At 24 h after transfection, cells were fixed with 4% paraformaldehyde and permeabilized by 0.25% Triton X-100. After blocking with 5% non-fat milk in PBS, the cell samples were incubated with anti-flag monoclonal antibody (Sigma, St. Louis, MO, USA) and then detected by IFKine Green conjugated goat anti-mouse polyclonal antibody (Abbkine, Redlands, CA, USA). Nuclear DNA was labeled with DAPI solution (Beyotime, Beijing, China); and (**C**) identification of the influence of eqMxA protein on influenza virus polymerase activity by influenza virus polymerase reconstitution assay. The pCAGGs-HLJ PB1 (50 ng), PB2 (50 ng), PA (50 ng), NP (100 ng), pRL-TK (10 ng), and pLW500-NPluci (100 ng) constructs, with a total amount of 750 ng of pCAGGs-eqMxA and a blank vector of pCAGGs (increasing amounts of 0 ng, 50 ng, 100 ng, 250 ng, 500 ng, and 750 ng of pCAGGs-eqMxA), were co-transfected into FEL cells in 48-well plates. At 24 h after transfection, relative luciferase activity was measured by the dual-luciferase assay. The relative luciferase activity subjected to 0 ng of pCAGGs-eqMxA was set as 100%. Each experiment was repeated three times. Statistical significance was compared with the result of the 0 ng pCAGGs-eqMxA group (**, *p* < 0.01; ***, *p* < 0.001).

To investigate the possible application of our cloned horse PolI promoter in studying influenza virus polymerase activity, the cDNAs of the PB1, PB2, PA, and NP segments of A/equine/Heilongjiang/SS1/2013 (HLJ/2013, H3N8 subtype EIV isolate of the American clade [28]) were cloned and inserted into pCAGGs (pCAGGs-HLJ PB2, PB1, PA, NP). Ten combinations of the pCAGGs plasmids encoding the PB1, PB2, PA, and NP proteins of Miami/1963 and HLJ/2013 (Figure 5A) were individually combined with pLW500-NPluci and pRL-TK and were co-transfected into FEL cells. The results of the dual-luciferase assay indicated that the polymerase activity of HLJ/2013 was approximately five times higher than that of Miami/1963 (*p* < 0.001). When each of the pCAGGs plasmid expressing the PB1, PB2, PA, and NP proteins of HLJ/2013 was replaced with that of Miami/1963, it was observed that the relative luciferase activity of the combinations decreased by approximately 40%–80% (*p* < 0.001), indicating that each of the PB1, PB2, PA, and NP proteins contributed to the higher polymerase activity of HLJ/2013. However, when each of the pCAGGs plasmids expressing the PB1, PB2, PA, and NP proteins of Miami/1963 was replaced with HLJ/2013, higher relative luciferase activity was found when the cells were subjected to NP and PA (*p* < 0.001), and lower relative luciferase activity was found when the cells were subjected to PB2 and PB1 (*p* < 0.001). This result suggested that the PB2 and PB1 proteins of HLJ/2013 were less compatible with the vRNPs associated proteins of Miami/1963 than the NP and PA proteins of HLJ/2013.

Both avian myxovirus resistance (Mx) protein and mammalian myxovirus resistance protein A (MxA) have been found to exert antiviral properties against the influenza virus [29,30,31]. Mx protein has been found to interact with vRNPs and disturb PB2-NP interactions, thus influencing viral polymerase activity [32]. The horse RNA PolI promoter cloned in our study was used in an EIV polymerase reconstitution assay to investigate whether the equine myxovirus resistance protein A (eqMxA) inhibits the viral polymerase activity. The eqMxA cDNA was cloned from FEL cells by RT-PCR (primers: CCG*GAATTC*atggttcattctgaagcgaa; CCG*CTCGAG*ttaacccgggaacttggctaacc; the restriction enzyme cutting sites of EcorI and XhoI are indicated in *italic*s), and then inserted into pCAGGs with an N-terminal Flag tag (DYKDDDDK) (pCAGGs-eqMxA). Like the human MxA and chicken Mx protein, the exogenous eqMxA protein was located in the cytoplasm, as determined in A549 and FEL cells by laser confocal scanning fluorescence detection (Figure 5B). Then, pCAGGs-HLJ PB1, PB2, PA, NP, pLW500-NPluci, and pRL-TK, together with increased amounts of pCAGGs-eqMxA, were co-transfected into FEL cells. The results showed that virus polymerase activity decreased when the transfection amount of eqMxA increased (Figure 5C), indicating that similar to the studied Mx protein, eqMxA also influence the influenza virus polymerase activity. However, the molecular mechanism how eqMxA inhibits the EIV polymerase activity still needs further study.

The PolI transcription initiation site of eukaryotes is usually identified by primer extension analysis [5,7]. However, studies on the PolI transcription initiation site of mammal species revealed that the −7, −5, −4, −1, and +2 to +8 positions of the PolI transcription initiation site were constant, whereas the −2 and +1 positions were A or G [6,7]. Via a homology search, the dog PolI transcription initiation site has been identified and applied to generate influenza virus in MDCK cells [6]. In our study, the horse PolI transcription initiation site was searched and identified by the same strategy. The homology characteristics of the sequence around the horse PolI transcription initiation site were completely in accordance with those of other mammal species (Figure 1B). Additionally, the transcription of the firefly luciferase located immediately downstream of the horse PolI promoter was indirectly proven by successfully detecting the firefly luciferase in the luciferase assay (Figure 3B and Figure 4). This result suggested that the horse PolI transcription initiation site was successfully identified using the homology search.

To date, no study has been performed on the changes in the polymerase activity of EIV since the pathogen was identified. In our study, the polymerase activity of HLJ/2013 was found to be approximately five times higher than that of Miami/1963 (Figure 5A), suggesting that compared with the RNPs of Miami/1963, the RNPs of HLJ/2013 were more adaptive to the host. In addition, it was noted that if the PA protein of HLJ/2013 was replaced with the PA protein of Miami/1963, the relative luciferase activity of the combination was equal to the activity of Miami/1963 (*p* > 0.05). If the PA protein of Miami/1963 was replaced with the PA protein of HLJ/2013, the highest relative luciferase activity was observed compared with PB1 (*p* < 0.001), PB2 (*p* < 0.001) and NP (*p* < 0.01). This result suggested that the PA protein played a more important role in the difference in polymerase activity between Miami/1963 and HLJ/2013. A comparison was made between the PA protein sequences of Miami/1963 and HLJ/2013, and a total of 36 residue mutations were identified. An asparaginic acid-to-asparagine change at residue 55 of the PA protein was observed between Miami/1963 and HLJ/2013, and has also been found in a large-scale comparative analysis of proteins, from avian and human strains [33]. However, the roles of residue 55 and the other residue sites of the PA protein in the host adaption when EIV evolves still need further study.

It has been suggested that the specific untranslated region of different segments of the influenza virus might influence the polymerase activity to different degrees, as identified in the human or avian influenza virus polymerase reconstitution assay [34,35]. In our study, only the specific, untranslated region of the NP segment of Miami/1963 is used in the EIV polymerase reconstitution assay. The following studies still need further investigation: if the specific untranslated region of the NP segment of Miami/1963 is replaced with that of other segments of Miami/1963, does the polymerase activity change? If the specific untranslated region of the NP segment of Miami/1963 is replaced with that of HLJ/2013 (the two strains have different 5′ and the same 3′ non-coding sequences of the NP segment), is the polymerase activity of HLJ/2013 still higher than that of Miami/1963? If the antisense firefly luciferase gene is flanked by the 5′ and 3′ non-coding sequences of other viral segment of Miami/1963 and HLJ/2013, does HLJ/2013 still have higher polymerase activity than Miami/1963?

## 3. Conclusions

In conclusion, the effective horse RNA PolI promoter sequence was cloned from horse cells, which works in the influenza virus polymerase reconstitution assay in horse cells. In addition, the polymerase activity of two EIV strains was compared by the developed polymerase reconstitution assay. Our study enriches our knowledge of the RNA PolI promoter in eukaryotic species, provides a useful tool for studying the polymerase activity of the influenza virus in horse cells, and may also contribute to the study of the host restriction factors blocking viral replication in horse cells.

## Supplementary Materials

The following are available online at www.mdpi.com/1999-4915/8/6/119/s1, Figure S1: Graphical representation of the construction strategy for pLW1000-NPluci, pLW750-NPluci, pLW500-NPluci, pLW250-NPluci, pLW125-NPluci, and pLW0-NPluci, Table S1: The detailed information on the primers used to construct pLW1000-NPluci, pLW750-NPluci, pLW500-NPluci, pLW250-NPluci, pLW125-NPluci, and pLW0-NPluci.
