# Supplementary material for: Cloning the Horse RNA Polymerase I Promoter and Its Application to Studying Influenza Virus Polymerase Activity
Source: Viruses. 2016 May 31;8(6):119. doi: 10.3390/v8060119 (PMC4926170; doi:10.3390/v8060119)
Supplement: Supplementary file 1 [file viruses-08-00119-s001.pdf]

Supplementary Materials: Cloning the Horse RNA Polymerase I Promoter and Its Application to Studying Influenza Virus Polymerase Activity

Gang Lu, Dong He, Zengchao Wang, Shudan Ou, Rong Yuan and Shoujun Li

The construction strategy for pLW1000-NPluci, pLW750-NPluci, pLW500-NPluci, pLW250-NPluci, pLW125-NPluci, and pLW0-Npluci:

The CMV promoter and human PolI promoter of pHW2000 were removed by primers ΔCMVPol-F and ΔCMVPol-R (Figure S1). The purified PCR product was named Seg1. Using the pGL3-Basic vector (Promega, Madison, WI, USA) containing the firefly luciferase coding sequence as the template, six PCR products were obtained by PCR with the same forward primer (Seg2-F) and six different reverse primers (Seg2-1000R, Seg2-750R, Seg2-500R, Seg2-250R, Seg2-125R, and Seg2-0R). These PCR products were named Seg2. Using the purified PCR product of the horse genomic DNA fragment amplified by primers 2146-F and 460-R as the template, five PCR products containing horse PolI promoters of different lengths were obtained by PCR with the same reverse primer (Seg2-R) and six different forward primers (Seg3-1000F, Seg3-750F, Seg3-500F, Seg3-250F, Seg3-125F, and Seg3-0F). These PCR products were named Seg3. Detailed primer information is listed in Table S1. The homologous arm was introduced between different segments by PCR with the designed primers to construct the target plasmid by homologous recombination (Figure S1). Plasmids pLW1000-NPluci, pLW750-NPluci, pLW500-NPluci, pLW250-NPluci, pLW125-Npluci, and pLW0-NPluci were constructed using the ClonExpress MultiS One Step Cloning Kit (Vazyme, Nanjing, China). For pLW1000-NPluci, pLW750-NPluci, pLW500-NPluci, pLW250-NPluci, and pLW125-NPluci, segments 1, 2, and 3 were used during the construction process. For pLW0-NPluci, only segments 1 and 2 were used during the construction process.

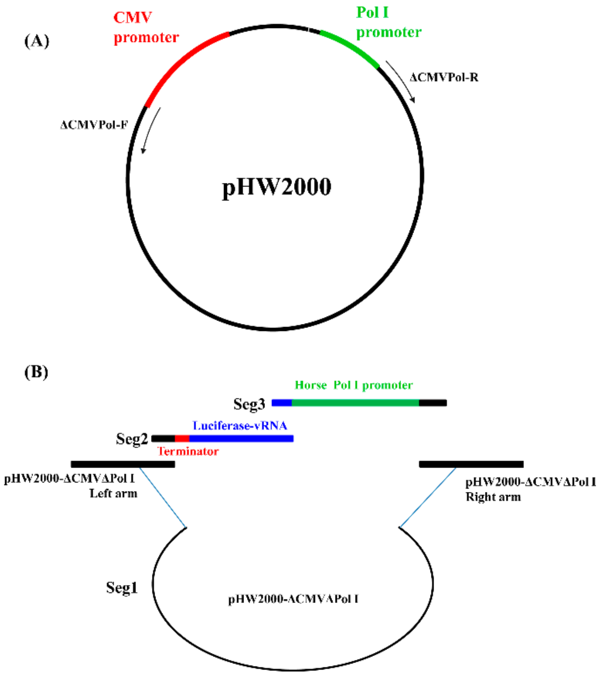

Figure S1. Graphical representation of the construction strategy for pLW1000-NPluci, pLW750-NPluci, pLW500-NPluci, pLW250-NPluci, pLW125-NPluci, and pLW0-NPluci. (**A**) The CMV promoter and human PolI promoter of pHW2000 were removed by primers ΔCMVPol-F and ΔCMVPol-R; (**B**) Plasmids pLW1000-NPluci, pLW750-NPluci, pLW500-NPluci, pLW250-NPluci, pLW125-NPluci, and pLW0-NPluci were constructed by homologous recombination. The homologous arm between different segments is indicated by the same color.

Table S1. The detailed information on the primers used to construct pLW1000-NPluci, pLW750-NPluci, pLW500-NPluci, pLW250-NPluci, pLW125-NPluci, and pLW0-NPluci.

Primer Name	Primer Sequence (5′→3′)
ΔCMVPol-F	AATGACCCCGTAATTGATTACTATTAATAA
ΔCMVPol-R	CACCTAAATGCTAGAGCTCGCTGATCAGCC
Seg2-F	AATTACGGGGTCATTCGGAGTACTGGTCGACCTCCGAAGTTGGG GGGGAGCAAAAGCAGGGTAGATAATCACTCACTGAGTGACATC AAAGCCATGGAAGACGCCAAAAACATAAAGA
Seg2-1000R	TGCCGCCAGATAAGTAGTAGAAACAAGGGTATTTTTCTTTACAC GGCGATCTTTCCGCCCTTC
Seg2-750R	CCGGCGACTCAGAGGAGTAGAAACAAGGGTATTTTTCTTTACAC GGCGATCTTTCCGCCCTTC
Seg2-500R	GGACCGGCCCCGGGAAGTAGAAACAAGGGTATTTTTCTTTACAC GGCGATCTTTCCGCCCTTC
Seg2-250R	AGGGCGGCCCGGTGGAGTAGAAACAAGGGTATTTTTCTTTACAC GGCGATCTTTCCGCCCTTC
Seg2-125R	GGGTGTGGGGCCTCCAGTAGAAACAAGGGTATTTTTCTTTACAC GGCGATCTTTCCGCCCTTC
Seg2-0R	TCTAGCATTTAGGTGAGTAGAAACAAGGGTATTTTTCTTTACAC GGCGATCTTTCCGCCCTTC
Seg3-1000F	ACTTATCTGGCGGCACAAAACCACCCATTC
Seg3-750F	CCTCTGAGTCGCCGGGTCAGGACTTAGAAAA
Seg3-500F	TCCCGGGGCCGGTCCACGCTCTTCTCCAGGG
Seg3-250F	CCACCGGGCCGCCCTCAGGACGACGGCCGGG
Seg3-125F	GGAGGCCCCACACCCCGGCCGGCACCGGGCG
Seg3-R	TCTAGCATTTAGGTGGGGATCTGGCCCGGTAGCGCCGGTGC

© 2016 by the authors; licensee MDPI, Basel, Switzerland. This article is an open access article distributed under the terms and conditions of the Creative Commons by Attribution (CC-BY) license (http://creativecommons.org/licenses/by/4.0/).
